# Semiconductor Nanowire Field-Effect Transistors as Sensitive Detectors in the Far-Infrared

**DOI:** 10.3390/nano11123378

**Published:** 2021-12-13

**Authors:** Mahdi Asgari, Leonardo Viti, Valentina Zannier, Lucia Sorba, Miriam Serena Vitiello

**Affiliations:** NEST, CNR-Istituto Nanoscienze and Scuola Normale Superiore, Piazza San Silvestro 12, 56127 Pisa, Italy; mahdi.asgari@nano.cnr.it (M.A.); leonardo.viti@nano.cnr.it (L.V.); valentina.zannier@nano.cnr.it (V.Z.); lucia.sorba@nano.cnr.it (L.S.)

**Keywords:** InAs nanowires, THz photodetectors, bolometeric effect, thermoelectric effect, Seebeck coefficient, room temperature

## Abstract

Engineering detection dynamics in nanoscale receivers that operate in the far infrared (frequencies in the range 0.1–10 THz) is a challenging task that, however, can open intriguing perspectives for targeted applications in quantum science, biomedicine, space science, tomography, security, process and quality control. Here, we exploited InAs nanowires (NWs) to engineer antenna-coupled THz photodetectors that operated as efficient bolometers or photo thermoelectric receivers at room temperature. We controlled the core detection mechanism by design, through the different architectures of an on-chip resonant antenna, or dynamically, by varying the NW carrier density through electrostatic gating. Noise equivalent powers as low as 670 pWHz^−1/2^ with 1 µs response time at 2.8 THz were reached.

## 1. Introduction

Photodetectors that operate in the terahertz (THz) frequency range (0.1–10 THz, wavelength range 3–3000 μm) have attracted increasing attention in the last two decades in a broad range of application fields, such as astronomy [1], spectroscopy [2], medicine [3], security [4], non-destructive quality testing [5] and, more recently, in quantum applications [6]. This has determined the proliferation of a variety of approaches encompassing different technologies between optics and microwave electronics, different physical mechanisms and several active material systems [7].

Key scientific directions at present include the following: the development of high-sensitivity (noise equivalent power NEP < 10^−19^ WHz^−1/2^) receivers operating at low temperatures for lab- [8] or space-based applications [9] (in this case they are often used as mixers in heterodyne schemes [1,10,11]) or quantum applications [6]; fast (response time τ < 1 ns) and sensitive (NEP < 10^−11^ WHz^−1/2^), room-temperature (RT), single-pixel devices (e.g., Schottky diodes [12], silicon MOSFETs [13], microbolometers [14]) for THz sensing and nanoimaging; and multi-pixel RT focal plane arrays [15,16] for tomography and industrial applications.

In the last decade, a broad research effort has been oriented towards the understanding of the relevant physical mechanisms that govern the detection of far-infrared light in low-dimensional materials. For example, low-dimensional materials, such as graphene [17,18,19,20], carbon nanotubes [21], graphene quantum dots (QD) [22,23] and other layered, two-dimensional (2D) materials [24], have been proposed and successfully demonstrated to operate as efficient THz-frequency receivers, once embedded in field-effect transistor (FET) architectures. Detection can be mediated by different effects [25], ranging from thermally-activated dynamics, e.g., bolometric [26] and photothermoelectric effects [19], to electronically-mediated rectification, e.g., resonant plasma-waves [27], photo-gating [22], photon-assisted tunneling [21], ballistic rectification [28] or through a combination of them.

Semiconductor nanowire (NW) FETs also represent a very promising platform for the development of THz-frequency light detectors, owing to their typically low shunt capacitance, in the order of few aF [29], and to the possibility of nano-engineering and fine-tuning fundamental material properties, such as chemical composition [30], doping [31] and morphology [32]. These are enabled by the degree of maturity achieved by the growth techniques: chemical beam epitaxy (CBE) and molecular beam epitaxy (MBE). Furthermore, thanks to recent advances in nano-manipulation techniques [33], semiconductor NWs can, in principle, be aligned in ordered arrays, opening the opportunity for device up scaling towards multi-pixel THz detectors [34].

Importantly, the versatility of the NW-FET platform allows it to be easily integrated in THz architectures with tailored detection properties [29,35,36], and to select, by design, the dominant physical mechanism underpinning THz detection. The detection dynamics in homogeneous InAs NWs have been investigated by THz scattering near-field optical microscopy (SNOM), in a recent work [37], which evidenced the interplay of two thermal effects ignited under photoexcitation: the bolometric effect (BE), activated at low carrier densities (*n*~10^16^ cm^−3^), and the photo-thermoelectric effect (PTE), activated at high carrier densities (*n* ≳ 10^17^ cm^−3^). The PTE effect has also been studied in quantum dots, defined in axially heterostructured InAs/InAs_0.3_P_0.7_ NWs [38] under excitation with photon energies lower than the inter-level spacing. Photodetection in an NW-QD is governed by the Seebeck effect, whose amplitude can be manipulated externally by applying an electrostatic gating to the QD. Furthermore, sub-micron sized NW-FETs can be integrated in sub-wavelength apertures for applications in coherent, high-resolution THz imaging and aperture-based, near-field optical microscopy (a-SNOM) [39].

In this work, taking advantage of the physical understanding of the core detection dynamics in semiconductor nanowire FETs, retrieved via near-field THz nanoscopy [37], we designed NW-based THz receivers where the activation of the BE and PTE could be selected by the antenna design and even dynamically tuned across a transition between the two mechanisms by simply changing the NW carrier density through electrostatic gating.

## 2. Materials and Methods

### 2.1. Bottom-Up Growth

**Figure 1:**
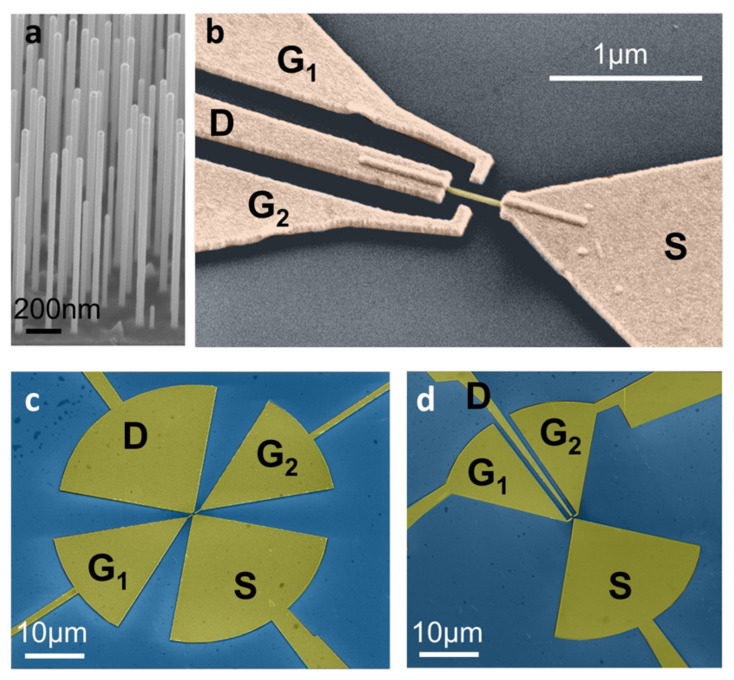
(**a**) 45° tilted scanning electron micrograph (SEM) image of the as-grown InAs NW forest. The scale bar corresponds to 200 nm. (**b**) False-colour SEM image of the fabricated lateral-gate NW-FET (asymmetric configuration). (**c**,**d**) False-colour SEM pictures of two NW-FETs in the s-FET (**c**) and a-FET (**d**) configurations.

Selenium-doped InAs NWs were grown by chemical beam epitaxy (CBE) in a Riber Compact-21 system by Au-assisted growth [40,41] on InAs (111)B-oriented substrates using trimethylindium (TMIn), tertiarybutylarsine (TBAs) and ditertiarybutyl selenide (DtBSe) as metal–organic (MO) pre-cursors. A thin (0.5 nm) Au film was deposited on the substrate at room temperature and Au nanoparticles were obtained upon annealing at 470 °C in the CBE chamber. The temperature was then decreased to 400 °C and the NW growth was started by funnelling onto the substrates the precursors TMIn, TBAs and DtBSe with line pressures of 0.3, 1.0 and 0.1 Torr, respectively. This allowed us to reach an electron carrier concentration of ~10^17^ cm^−3^ in the InAs NWs. Selenium incorporation has been demonstrated to improve NW mobility and contact resistance [40,42]. The as-obtained NWs had a wurtzite crystal structure, a ~1.5 μm length and diameters ranging from 30 to 50 nm (Figure 1a).

### 2.2. Device Design and Fabrication

Homogeneous Se-doped InAs NWs were integrated within lateral dual gated FETs, as shown in Figure 1b. NWs were first transferred through a mechanical dry-transfer approach from the growth substrate over a 300 nm/350 μm SiO_2_/intrinsic silicon host wafer. Subsequently, source (S), drain (D) and gate (G) electrodes were defined by means of an aligned electron-beam lithography (EBL) procedure, Cr/Au (10/100 nm) thermal evaporation and lift-off. The detectors were then mounted and wire bonded on dual inline packages.

In order to activate different detection mechanisms, we designed two distinct device geometries: a symmetric FET configuration (s-FET, Figure 1c), where the S and D electrodes were connected to the two arms of a planar, bow-tie antenna, and an asymmetric configuration (a-FET, Figure 1b,d), where the S and G electrodes were connected to the antenna arms, with the D electrode defined as a thin wire between the dual lateral gates. The total length of the bow-tie antennas (L = 44 μm) was chosen after electromagnetic simulations, performed with a finite-element method (FEM) in a commercial software (COMSOL Multiphysics, version 5.0, COMSOL, Burlington, MA, USA) [18].

In s-FETs, the THz energy was symmetrically driven to the NW, whose temperature was expected to rise homogeneously as a consequence of free-carrier absorption [43]. Instead, in a-FETs, the bow-tie produced a THz-induced field enhancement at the S-side of the NW, generating a thermal gradient along it. Therefore, the two configurations should, in principle, have favored the onset of different physical mechanisms [37]; the BE in the s-FET and the PTE in the a-FET.

## 3. Results

### 3.1. Electrical Characterization

**Figure 2:**
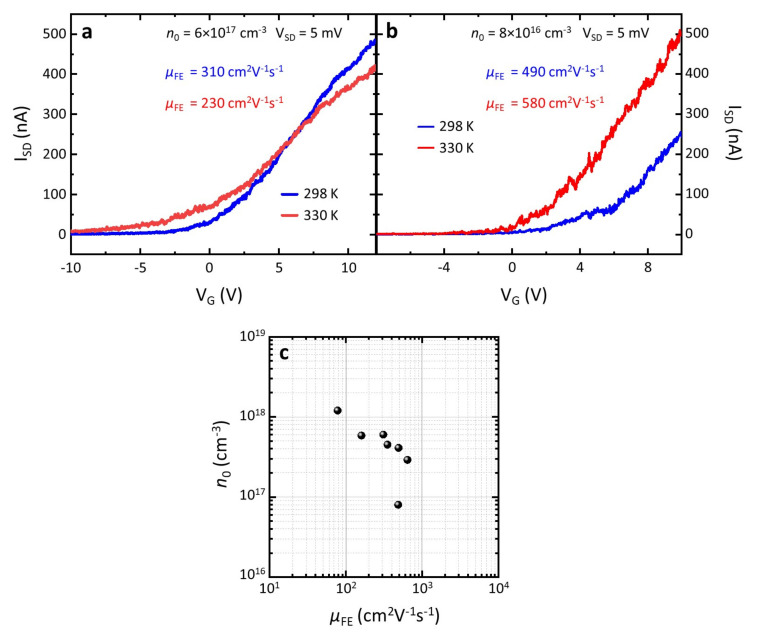
(**a**,**b**) I_SD_ vs. V_G_ curve collected at V_SD_ = 5 mV at two distinctive temperatures, 298 K and 330 K, measured in the s-FET (**a**) and in the a-FET (**b**) with *n*_0_ = 6 × 10^17^ cm^−3^ (**a**) and *n*_0_ = 8 × 10^16^ cm^−3^ (**b**). (**c**) Chart of *n*_0_ vs. *μ*_FE_: every point corresponds to a different device.

The transport characteristics of the designed NW-FETs were measured with two *dc* voltage generators (Keithley2400, Tektronix, Beaverton, OR, USA) to drive the source-drain bias (V_SD_) and gate voltage (V_G_, kept identical for G_1_ and G_2_), while monitoring the current (I_SD_) through the NW channel. In the employed electrical setup, the heat sink temperature (T) could be set and monitored by the combination of a heater and a temperature sensor, which allowed the control of the device temperature during operation. Figure 2a,b shows the I_SD_ vs. V_G_ curve collected for the two samples in the two different antenna configurations, recorded at two different temperatures, 298 K and 330 K. We then estimated the transconductance (*g*) curve as the first derivative of the I_SD_ vs. V_G_ characteristic, from which we could retrieve the field-effect mobility (*μ*_FE_) and the pristine carrier density (*n*_0_) of the individual NWs. Indeed, *μ*_FE_ could be estimated from the maximum transconductance (*g*_m_) using the formula [36] *μ*_FE_ = *g*_m_*w*_G_^2^/(C_wG_V_SD_), where *w*_G_ is the gate width and C_wG_ is the gate-to-channel capacitance; *n*_0_ could be calculated as *n*_0_= C_wG_V_th_/(*e*πr^2^*w*_G_) [36], where V_th_ is the NW-FET threshold voltage, *e* is the elementary charge and πr^2^ is the cross-sectional area of the NW, approximated as a cylinder. Importantly, the estimation of both parameters required knowledge of C_wG_. For the two different architectures, C_wG_ was simulated using a commercial software (COMSOL Multiphysics, version 5.0, COMSOL, Burlington, MA, USA). We obtained C_wG_ = 12.3 aF for the *s*-FET and C_wG_ = 18.1 aF for the *a*-FET.

Figure 2a,b also shows that, for samples with *n*_0_ ≳ 5 × 10^17^ cm^−3^, *μ*_FE_ decreased with increasing T and at large and positive values of V_G_. The conductivity (*σ*) decreased with increasing T, i.e., beyond a specific V_G_ the NW behaved as a degenerate semiconductor. For samples with *n*_0_ ≲ 5 × 10^17^ cm^−3^, *μ*_FE_ was instead slowly varying with T. In both cases, *n*_0_ increased with T as a consequence of the thermal activation of surface donors [44].

Figure 2c presents a scatter plot of the as-obtained values of *μ*_FE_ and *n*_0_ for the devised NW-FETs, at 298 K. *μ*_FE_ ranged from 80 to 650 cm^2^ V^−1^s^−1^, while *n*_0_ ranged from 0.8 to 20 × 10^17^ cm^−3^. This latter spread in the carrier density was a combined effect of ambient pressure exposure, processing-related factors and different nanowire morphologies (e.g., diameter).

### 3.2. Antenna Characterization

**Figure 3:**
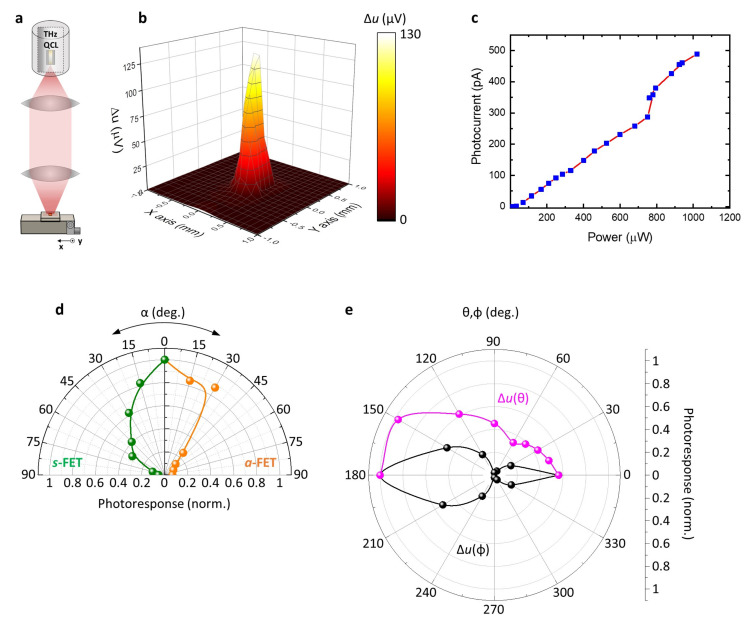
(**a**) Schematic of the experimental setup for optical characterization. (**b**) Intensity profile measured with an s-FET in the focal point. (**c**) Dependence of the detector’s photocurrent from the input optical power. (**d**) Polar plot of the normalized photoresponse, recorded as a function of the angle (α) between the light polarization and antenna axis for symmetric (green) and asymmetric (orange) antennas. (**e**) Antenna radiation pattern measured as a function of azimuth angle (φ, black dots) and elevation angle (θ, magenta dots). The direction (θ = 0°, φ = 0°) was pointing out of the substrate, perpendicularly to the antenna plane.

The optical characterization of the investigated NW-FETs was performed by employing the experimental setup shown in Figure 3a. A linearly polarized 2.8 THz wave was generated by a quantum cascade laser (QCL), refrigerated at a heat sink temperature of 30 K by a Stirling cryocooler (Ricor K535, Ein Harod, Israel) and operated in pulsed mode (40 kHz repetition rate, duty cycle 4%), capable of delivering to the detector a maximum optical power (average) P_o_ ~1.1 mW, calibrated with a power meter (Ophir 3A-P-THz). The divergent QCL beam was first collimated by a TPX (polymethylpentene polymer) lens with 1′ focal length and then focused by a TPX lens with 2′ focal length in a circular focal spot of radius ~200 μm (evaluated as full width at half maximum), as retrieved by the measured beam profile in the focal point (Figure 3b). The QCL power in the focal point was set to P_o_ = 400 μW, which corresponded to a THz intensity I_o_ = 0.32 Wcm^−2^. The detectors were mounted on an *xyz* motorized stage and a rotational stage was employed to manually adjust the polarization (α), azimuthal (φ) and elevation (θ) angles.

The measured photovoltage (Δ*u*), recorded at the D electrode while keeping S grounded, was then pre-amplified with a voltage preamplifier (DL Instruments, model M1201 Brooktondale, NY, USA, gain G = 1000) and sent to a lock-in (SR5210). We used as lock-in reference a modulation frequency of 1.333 kHz, which was also used as a square-wave envelope for the QCL pulses. Δ*u* could be inferred from the lock-in reading (V_LI_) via the relation Δ*u* = (π√2/2) × V_LI_/G [29], where the pre-factor π√2/2 took into account that the lock-in measured the root mean square of the fundamental sine wave Fourier component of the square wave [45] produced by the QCL modulation. Figure 3c shows the dependence of the photocurrent recorded with one of the NW-FET detectors as a function of P_o_, demonstrating the NW-FET linearity.

We then characterized the response of the two antenna configurations with respect to the polarization angle, by measuring Δ*u* while the sample was rotated around the beam propagation direction on the antenna plane. The polarization responses for the s-FET and a-FET are reported in Figure 3d: in both cases, the signal was at its maximum when the antenna axis was parallel to the THz electric field (α = 0°).

We evaluated the antenna directivity *D*_0_ by recording Δ*u* as a function of the angles φ (H-plane) and θ (E-plane). Figure 3e shows the results retrieved with an a-FET. The antenna directivity in a given direction (θ,φ) was defined as the ratio between the antenna radiation intensity in that direction and the radiation intensity averaged over all directions. The directivity was therefore evaluated in the direction orthogonal to the antenna surface, which pointed out of the silicon substrate (θ,φ) = (0,0) as *D*_0_ = Δ*u*(0,0)/⟨Δ*u*(θ,φ)⟩ = 3.75, where ⟨…⟩ represents the average photovoltage over an angle of 4π, calculated as a series approximation, assuming that the variations over θ and φ as separable [46].

### 3.3. Optical Characterization

One of the most important figures of merit for a THz photodetector is the voltage responsivity (R_v_), defined as the ratio between Δ*u* and the optical power (P_a_) impinging on the detector. P_a_ is related to the intensity in the focal point through the detector effective area (A_eff_), as P_a_ = I_o_ × A_eff_. We calculated A_eff_ as the diffraction limited area [47] A_eff_ = λ^2^/4 = 2800 μm^2^, where λ is the free-space wavelength. We note that, from the knowledge of *D*_0_, it is possible to evaluate the effective area using a different formalism [48]: A_eff_ = *D*_0_λ^2^/4π = 3300 μm^2^. Thus, in the present geometry, there is a <20% difference in the estimation of A_eff_ between the diffraction-limited method and the antenna-directivity method.

**Figure 4:**
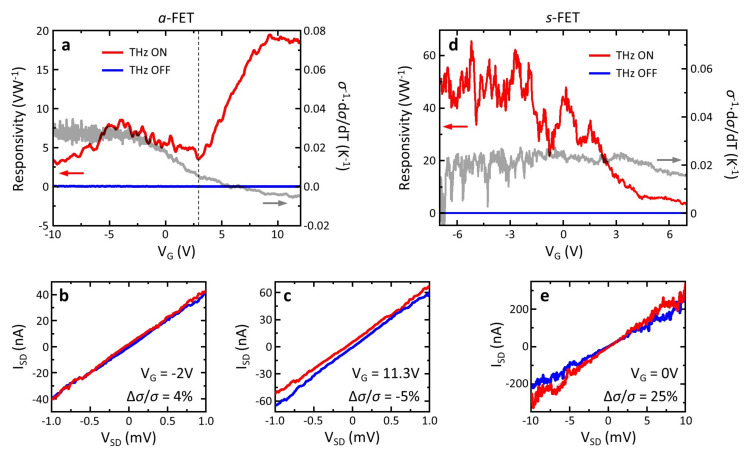
(**a**) Left vertical axis: R_v_ measured as a function of V_G_, in a prototypical a-FET; the blue line showed the noise level when the THz beam was blanked. Right vertical axis: expected trend of Δ*u*_B_(V_G_), calculated from the transconductance characteristics, measured at different heat sink temperatures. The dashed vertical line indicated the value of V_G_ > 3 V, where the PTE contribution started to dominate the photoresponse. (**b**,**c**) I_SD_ vs. V_SD_ traces recorded for the a-FET in the illuminated (red) and dark (blue) states at different V_G_. (**d**) Left vertical axis: R_v_(V_G_) measured for an s-FET. Right vertical axis: expected trend of Δ*u*_B_(V_G_). (**e**) I_SD_ plotted as a function of V_SD_, measured at V_G_ = 0 V. All the measurements were collected at room temperature.

The plot of R_v_ as a function of V_G_ for the investigated *a*-FETs (Figure 4a), displayed a signal-to-noise ratio SNR > 600. The R_v_ curve showed a non-monotonic trend, which we ascribed to the non-trivial interplay of the BE and PTE mechanisms. The bolometric photovoltage Δ*u*_B_ is expected to be proportional to the quantity *β*/*σ*, where *σ* was the static conductivity and *β* ≡ d*σ*/dT is the bolometric coefficient, which quantifies the sensitivity of the electrical conductivity with respect to a temperature change [24]. Thus, from the measurement of *σ*(V_G_) at different temperatures, it is possible to extrapolate *β*(V_G_). The expected trend of Δ*u*_B_(V_G_) is reported in Figure 4a as a grey curve overlaid on R_v_(V_G_). Interestingly, the bolometric effect could explain the photoresponse only for V_G_ < 3 V, whereas at higher V_G_ another physical mechanism seemed to dominate. To verify this conclusion, we extrapolated the detector photocurrent by quantifying the change of the I_SD_ vs. V_SD_ characteristic between the illuminated state (THz-on) and the dark state (THz-off), while keeping S grounded and while sweeping V_SD_. At V_G_ = −2 V (Figure 4b), the THz-on and THz-off traces almost overlapped, and the effect of THz radiation was visible only as a positive change of the NW conductivity Δ*σ*/*σ* = 4%, i.e., an increase in *σ* when the system was heated by the THz beam. At V_G_ = 11.3 V, instead, there was a rigid shift of the I_SD_ vs.V_SD_ characteristic towards positive currents, as a consequence of an additional electromotive force along the channel, which pushed electrons from S to D. We ascribed this contribution to the PTE-driven photocurrent: I_PTE_ = −*σ*S_b_∇T, where S_b_ is the NW Seebeck coefficient and ∇T is the THz-induced (positive) thermal gradient between the D (cold) and S (hot) electrodes, resulting in I_SD_ = *σ*(V_SD_–S_B_∇T) [38] (here a positive V_SD_ corresponds to a negative electrostatic voltage gradient from D to S). Importantly, for V_G_ > 3 V, the BE was still observable in a negative Δ*σ*/*σ* = −5%, in agreement with the expected trend of Δ*u*_B_(V_G_).

We note that the experimentally measured responsivity departed from the expected bolometric response for V_G_ < −6 V. This discrepancy can be attributed to two main factors: (i) the loading effect, which is a general phenomenon that affects the responsivity of FETs [48] and (ii) the hysteresis in the NW-FET transport characteristics [49], which makes it difficult to replicate the initial conditions of consecutive V_G_ sweeps, especially if the NW is operated at ambient pressure and temperature.

A different behaviour was observed in *s*-FETs (Figure 4d). In this case, R_v_ decreased as a function of V_G_, qualitatively following the trend of Δ*u*_B_(V_G_) over the whole gate voltage sweep. This indicated that in symmetric architectures, the photoresponse was mainly driven by the BE. Figure 4e shows the variation of the I_SD_ vs. V_SD_ characteristic between the illuminated and dark states, testifying a huge change in the NW conductivity upon illumination, Δ*σ*/*σ* = 25%.

### 3.4. Detector Performance

**Figure 5:**
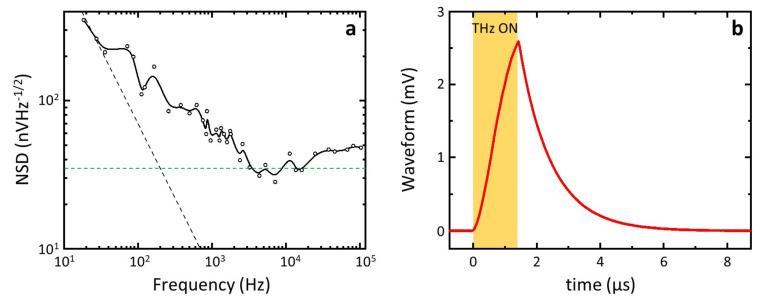
(**a**) NSD measured with a lock-in oscillator frequency sweep technique. The dashed black line represented the 1/*f* (or flicker noise) term, and the dashed green line represented the thermal noise floor. (**b**) Detection signal recorder with an a-FET at V_G_ = 12 V, with a 5.0 GS/s oscilloscope, showed a response time of *τ*∼1 μs. The QCL pulse duration was set to 1.6 μs, corresponding to the yellow-shaded area.

To assess the detector sensitivity, we evaluated NEP as the ratio between the noise spectral density (NSD) and R_v_. We measured the root mean square of NSD by connecting the detectors to a lock-in amplifier and employing an internal oscillator frequency (*f*) sweep technique [50]. The amplitude of the as-measured NSD was reported in Figure 5a for the a-FET (sample corresponding to Figure 2a and Figure 4a). The noise figure was dominated by the flicker noise for *f* < 4 kHz, whereas it flattened close to the thermal noise floor (Johnson-Nyquist noise N_J_) at higher frequencies. At the modulation frequency employed in our experiments *f* = 1.333 kHz, the NSD was ~2 N_J_ = 2 × (4 k_B_RT)^1/2^, where k_B_ is the Boltzmann constant and R is the NW resistance. From the knowledge of NSD we found a minimum NEP = 2 nWHz^−1/2^ among the tested a-FETs and a minimum NEP = 670 pWHz^−1/2^ among the tested s-FETs.

Finally, we characterized the THz detection speed of the NW-FETs by recording the time trace of the photovoltage with a fast oscilloscope. For this measurement, we used a THz pulse duration of 1.6 μs and we connected the detector output (D electrode) to a high-bandwidth (200 MHz) voltage preamplifier (Femto, HVA-200M-40-F) before the oscilloscope. The recorded waveform is depicted in Figure 5b. The waveform discharge ramp was then fitted with the equation [18] V_out_ = P_1_ + V_peak_ × exp(−(*t*−P_2_)/*τ*), where V_out_ is the voltage read by the oscilloscope, *t* is the time independent variable, P_1_, V_peak_ and P_2_ are fitting parameters and *τ* was the detector response time. In the whole batch of samples, we extracted *τ*~1 μs.

## 4. Conclusions

We engineered semiconductor nanowire field-effect transistors operating as sensitive bolometers or phototermoelectric receivers at 2.8 THz, at room temperature. We selected and controlled the dominant detection dynamics via the symmetry of a lithographically patterned on-chip resonant antenna and through electrostatic gating, respectively. The devised detectors showed state-of-the-art room temperature noise equivalent powers (0.67–2 nWHz^−1/2^) and response times of (1 µs), suitable for real-time sensing, security and imaging applications in the far infrared, opening realistic perspectives for the development of potential nanoarrays for multi-pixel image reconstruction at high (>2 THz) THz frequencies.

## Data Availability

The data presented in this study are available on reasonable request from the corresponding author.
